# Supplementary material for: Challenges in Imaging Analyses of Biomolecular Condensates in Cells Infected with Influenza A Virus
Source: Int J Mol Sci. 2023 Oct 17;24(20):15253. doi: 10.3390/ijms242015253 (PMC10607852; doi:10.3390/ijms242015253)
Supplement: Supplementary file 1 [file ijms-24-15253-s001.zip › ijms-2570089 supplementary material.pdf]

International Journal of *Molecular Sciences*

MDPI

Supplementary Material

Challenges in Imaging Analyses of Biomolecular Condensates in Cells Infected with Influenza A Virus

Temitope Akhigbe Etibor [1,†], Aidan O'Riain [1,†], Marta Alenquer [1,2], Christian Diwo [1], Sílvia Vale-Costa [1] and Maria João Amorim [1,2,*]

The supplementary materials contain 3 supplementary figures, 3 tables and figure legends for 4 videos.

Supplementary Figure S1

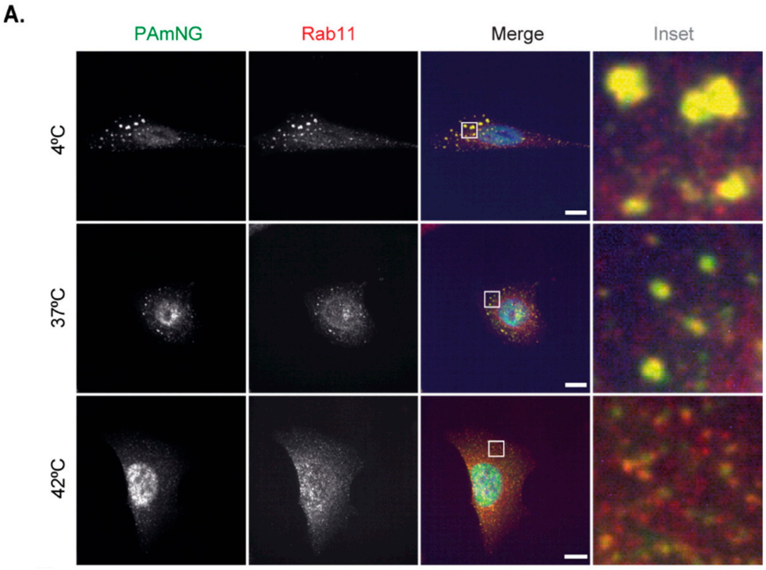

Supplementary Figure S1 – Liquid viral inclusions produced by the PA-mNG PR8 virus react to temperature.

A549 were infected at an MOI of 10 incubated at different temperatures (4ºC, 37ºC, 42ºC) for 30 min (E), fixed 16hpi, and analysed by immunofluorescence using antibody staining against Rab11 and NP as a proxy for vRNP. Scale bar = 10 µm.

Supplementary Figure S2

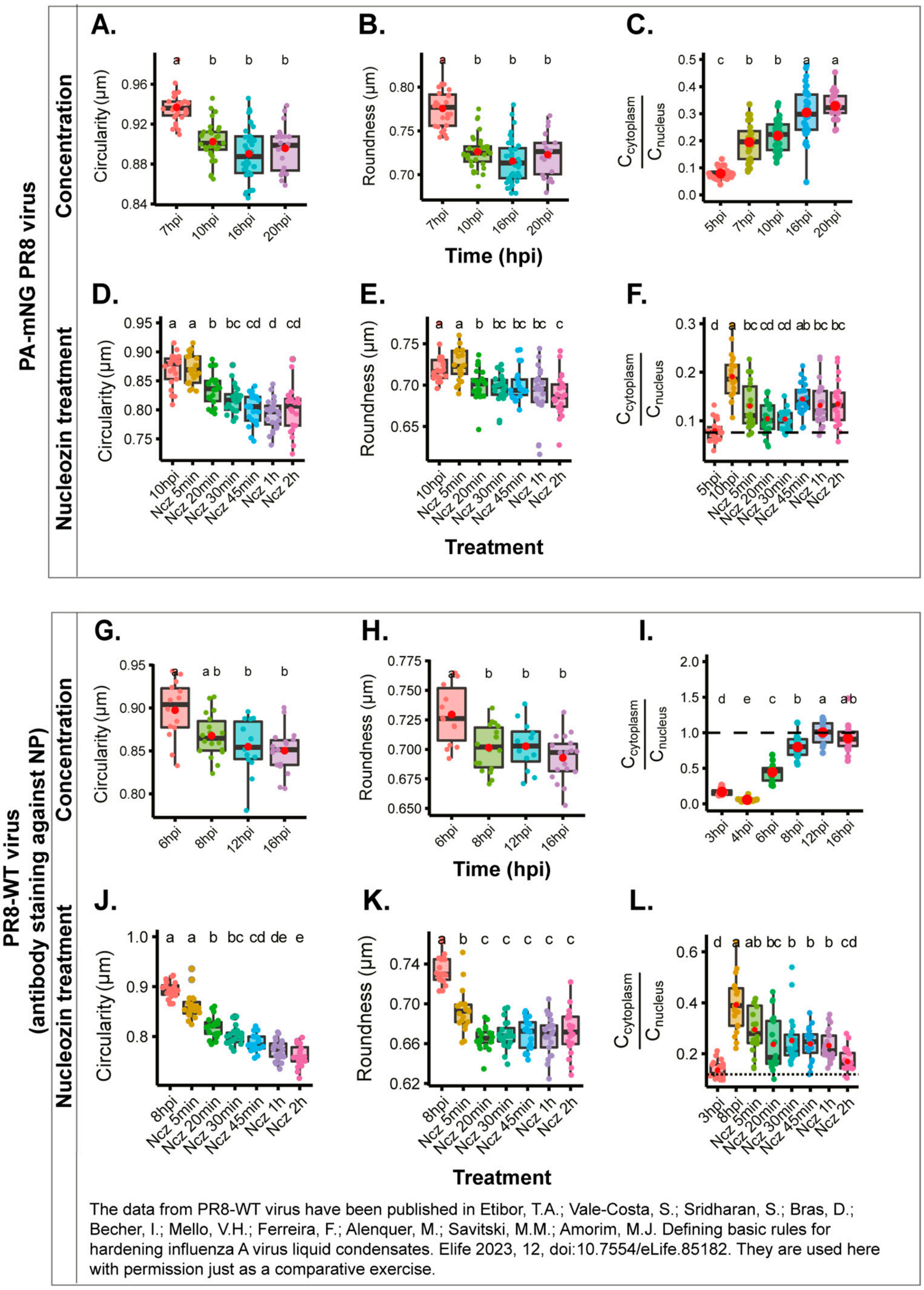

Supplementary Figure S2. – Biophysical traits obtained with Z-projected images vary in PA-mNG PR8 and PR8-WT infected cells. Biophysical calculations in cells infected with the PA-mNG PR8 virus (A-F) or PR8-WT virus (G-L) upon altering the concentration (A-C, G-I) by taking advantage of the time course of infection as indicated or upon altering the type/strength of vRNP interactions by adding 5µM of (Ncz) at 10 hpi during the indicated time periods (D-F, J-L). All data: circularity, roundness and cytoplasm/ nucleus concentration ratio are represented as boxplots. Above each boxplot, same letters indicate no significant difference between them, while different letters indicate a statistical significance at $\alpha = 0.05$ using Kruskal-Wallis Bonferroni treatment for non-parametric analysis. The data of PR8-WT virus have been published in [30] and are only used here for comparative terms. (A, D, G, J). Boxplot of viral inclusion circularity per cell; statistical data by Kruskal Wallis Bonferroni treatment. (B, E, H, K). Boxplot of roundness of inclusions; statistical data by Kruskal Wallis Bonferroni treatment. (C, F, I, L) Boxplot of cytoplasm concentration of vRNPs divided by the nuclear concentration of vRNPs; statistical data by Kruskal Wallis Bonferroni treatment.

Supplementary Figure S3

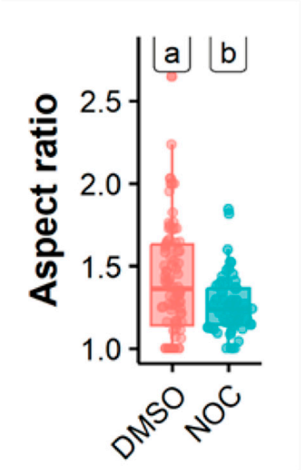

Supplementary Figure S3 – Treatment with nocodazole reduces the aspect ratio of influenza A virus inclusions.

Cells (A549) were infected with PR8 WT for 8 h and treated with nocodazole (10 µg/mL) for 2h, after which the movement of influenza A virus inclusions was captured by live cell imaging. Viral inclusions were segmented, and the aspect ratio measured by imageJ, analysed and plotted in R.

Supplementary Table S1. – Summary of the biophysical and topological traits for PA-mNG viruses under the different conditions tested for comparative purposes (Mean ± SD).

	Analyses	Ccytoplasm/ Cnucleus ± SD	Area (μm²) ± SD	Aspect ratio ± SD	C(dense) (AU) ± SD	C(dilute) (AU) ± SD	ΔΔG ($J.mol^{-1}$) ± SD	N° of inclusions ± SD	Circularity ± SD	Roundness ± SD
One focal plane - 10 hpi + 2h DMSO	Bottom CM	0.64 ± 0.2	0.28 ± 0.1	1.49 ± 0.1	20.75 ± 6.7	6.30 ± 2.3	-3033.0 ±920	151.6 ± 63	0.90 ± 0.02	0.73 ± 0.03
	Middle CM	0.48 ± 0.2	0.33 ± 0.1	1.50 ± 0.1	22.07 ± 7.54	5.95 ± 2.2	-3465.4 ± 996	96.8 ± 58	0.89 ± 0.03	0.72 ± 0.03
	Top CM	0.40 ± 0.1	0.28 ± 0.1	1.56 ± 0.1	11.49 ± 8.1	4.98 ± 2.4	-2000.4 1149±	119.3 ± 90	0.87 ± 0.04	0.71 ± 0.04
One focal plane - 10 hpi + 2h nucleozin	Bottom NCZ	0.51 ± 0.2	0.53 ± 0.1	1.46 ± 0.1	37.73 ± 17.1	4.82 ± 4.7	-4727.4 ± 828	55.5 ± 28	0.90 ± 0.04	0.74 ± 0.02
	Middle NCZ	0.53 ± 0.2	0.66 ± 0.3	1.48 ± 0.1	51.25 ± 18.0	6.00 ± 5.2	-5180.1 ± 943	57.0 ± 33	0.90 ± 0.04	0.74 ± 0.01
	Top NCZ	0.46 ± 0.2	0.72 ± 0.3	1.49 ± 0.2	54.24 ± 20.6	6.18 ± 6.2	-5358.6 ± 661	39.5 ± 24	0.90 ± 0.05	0.73 ± 0.01
Z projection - time course of infection	CM 5h	0.21 ± 0.1	N/A	N/A	N/A	29.97 ± 58.6	N/A	N/A	N/A	N/A
	7h	0.34 ± 0.1	0.21 ± 0.03	1.37 ± 0.05	603.09 ± 236.0	165.40 ± 125.4	-2325.2 ± 1442	231.5 ± 127	0.94 ± 0.02	0.78 ± 0.02
	10h	0.36 ± 0.1	0.29 ± 0.04	1.47 ± 0.05	817.58 ± 361.0	291.65 ± 182.9	-2326.4 ± 507	216 ± 94	0.90 ± 0.02	0.73 ± 0.02
	16h	0.46 ± 0.1	0.36 ± 0.1	1.49 ± 0.1	1576.45 ± 637.8	575.43 ± 412.4	-2443.4 ± 356	177.5 ± 86	0.89 ± 0.03	0.72 ± 0.03
	20h	0.47± 0.1	0.39 ± 0.1	1.47 ± 0.1	1559.90 ± 960.8	464.69 ± 633.8	-2757.4 ± 931	156 ± 63	0.90 ± 0.02	0.72 ± 0.02
Z projection – addition of nucleozin	10hpi	0.18 ± 0.04	0.46 ± 0.2	1.47 ± 0.04	388.1 ± 185	149.23 ± 73	-1972.3 ± 252	298.4 ± 168	0.87 ± 0.03	0.72 ± 0.02
	NCZ 5 min	0.11 ± 0.04	0.509 ± 0.2	1.46 ± 0.04	504.8 ± 169	133.84 ± 62	-3108.1 ± 455	286.3 ± 234	0.87 ± 0.03	0.72 ± 0.02
	NCZ 30 min	0.10 ± 0.03	1.23 ± 0.3	1.51 ± 0.05	1104.7 ± 302	125.41 ± 50	-4543.4 ± 621	77.8 ± 84	0.82 ± 0.02	0.70 ± 0.02
	NCZ 45 min	0.14 ± 0.03	1.34 ± 0.4	1.53 ± 0.05	929.3 ± 260	161.52 ± 77	-3763.1 ± 581	82.7 ± 70	0.80 ± 0.03	0.70 ± 0.02
	NCZ 2h min	0.13 ± 0.05	1.34 ± 0.51	1.55 ± 0.07	834.8 ± 463	133.81 ± 94	-4344.1 ± 882	50.2 ± 43	0.80 ± 0.04	0.69 ± 0.03

Supplementary Table S2. – Summary of the biophysical and topological traits for PR8-WT tested for Z-protections under the two conditions tested: time course and adding nucleozin.

	Analyses	Ccytoplasm/ Cnucleus ± SD	Area (µm²) ± SD	Aspect ratio ± SD	C(dense) (AU) ± SD	C(dilute) total (AU) ± SD	ΔΔG (J.mol⁻¹) ± SD	N° of inclusions ± SD	Circularity ± SD	Roundness ± SD
Z projection - time course of infection	CM 3h	0.29 ± 0.05	N/A	N/A	448.4 ± 108.3	255.1 ± 64.9	0 ± 503.5	0 ± 33.1	N/A	N/A
	CM 4h	0.10 ± 0.03	N/A	N/A	478.49 ± 164.1	283.1 ± 61.8	-457.74 ± 893.2	0 ± 47.1	N/A	N/A
	CM 6h	1.56 ± 0.13	0.17 ± 0.05	1.45 ± 0.07	23566.26 ± 5940.9	6721.70 ± 2463.5	-1799.64 ± 623.1	191.21 ± 178.1	0.90 ± 0.03	0.73 ± 0.03
	CM 8h	2.31 ± 0.15	0.24 ± 0.09	1.51 ± 0.05	39680.94 ± 12407.2	14447.85 ± 6376.29	-1139.75 ± 382.2	230.56 ± 193.7	0.87 ± 0.03	0.70 ± 0.02
	CM 12h	2.95 ± 0.16	0.25 ± 0.06	1.55 ± 0.16	34718.93 ± 13066.9	13692.58 ± 3826.05	-1131.23 ± 444.1	114.5 ± 102	0.85 ± 0.03	0.68 ± 0.05
	CM 16h	2.06 ± 0.19	0.29 ± 0.06	1.52 ± 0.05	28094.89 ± 6657.48	12394.27 ± 2860.9	-833.77 ± 342.4	143 ± 97.8	0.85 ± 0.03	0.69 ± 0.02
Z projection – addition of nucleozin	3hpi	0.19 ± 0.03	N/A	N/A	29.75 ± 7.71	19.7 ± 5.9	0 ± 405.5	0 ± 0	N/A	N/A
	8hpi	1.03 ± 0.1	0.28 ± 0.04	1.43 ± 0.03	2091.66 ± 417.65	728.12 ± 213	-1711.07 ± 396.6	310.5 ± 133.7	0.89 ± 0.02	0.73 ± 0.01
	NCZ 5 min	1.26 ± 0.09	0.35 ± 0.07	1.52 ± 0.06	1946.9 ± 443.1	481 ± 155.2	-2870.16 ± 724.8	235 ± 104.7	0.86 ± 0.03	0.69 ± 0.02
	NCZ 20 min	1.36 ± 0.1	0.47 ± 0.08	1.59 ± 0.03	2349.44 ± 449.2	324.2 ± 97.2	-3956 ± 663.4	150.5 ± 65.7	0.82 ± 0.02	0.67 ± 0.01
	NCZ 30 min	1.37 ± 0.11	0.57 ± 0.12	1.58 ± 0.04	2555.37 ± 454.13	296.51 ± 113.5	-4467.91 ± 820.8	114 ± 81.17	0.80 ± 0.02	0.67 ± 0.01
	NCZ 45 min	2.07 ± 0.07	0.68 ± 0.11	1.58 ± 0.05	3094.13 ± 391.8	336.1 ± 90.9	-4545.54 ± 719.7	88.5 ± 49	0.79 ± 0.02	0.67 ± 0.02
	NCZ 1h	2.15 ± 0.06	0.78 ± 0.14	1.58 ± 0.05	2956.6 ± 558.7	356.9 ± 116.8	-4813.17 ± 674.8	66 ± 54.1	0.77 ± 0.02	0.67 ± 0.02
	NCZ 2h	1.88 ± 0.05	1.02 ± 0.18	1.56 ± 0.06	3569.45 ± 640	298.61 ± 94	-5388.44 ± 808.5	38 ± 34	0.76 ± 0.02	0.67 ± 0.02

Supplementary Table S3. – Summary and comparison of the biophysical and topological traits for IAV inclusions using Imaris software (considering cellular volume), 10 hours post-infection, adding nucleozin from 10 hpi. Comparisons show different detection modalities, namely: tagged virus, anti-mNeonGreen monoclonal antibody, and anti-Nucleoprotein antibody (Mean ± SD).

	**Analyses**	**Area (μm²) ± SD**	**Sphericity ± SD**	**C(dense) (AU) ± SD**	**C(dilute) total (AU) ± SD**	**ΔG (J.mol⁻¹) ± SD**	**N° of inclusions ± SD**	**Volume (μm³) ± SD**
3-dimensional analysis – treatment conditions and labelling used	3D CM 10h – PA-mNG	1.04 ± 1.02	0.83 ± 0.04	229.51 ± 58.18	140.91 ± 17.41	-1185.14 ± 442.32	319.56 ± 120.9	0.097 ± 0.14
	3D NCZ 2h – PA-mNG	2.94 ± 0.73	0.88 ± 0.02	451.4 ± 77.95	210.37 ± 74.28	-1994.01 ± 460.14	164.24 ± 71.2	0.5 ± 0.18
	3D CM 10h – anti-mNeonGreen	0.56 ± 0.14	0.83 ± 0.02	219.67 ± 52.59	154.67 ± 23.81	-733.88 ± 391.56	365.31 ± 142.2	0.037 ± 0.12
	3D NCZ 2h – anti-mNeonGreen	3.31 ± 1.01	0.86 ± 0.03	411.84 ± 223.37	215.5 ± 93.64	-1532.17 ± 790.26	182.72 ± 89.3	0.57 ± 0.22
	3D CM 10h – anti-Nucleoprotein	0.70 ± 0.18	0.86 ± 0.01	209.17 ± 46.11	152.86 ± 24.13	-697.73 ± 204.87	448.75 ± 117.3	0.051 ± 0.02
	3D NCZ 2h - anti-Nucleoprotein	2.91 ± 0.85	0.86 ± 0.03	427.75 ± 147.61	211.46 ± 65.15	-1638.89 ± 495.75	189.4 ± 97.5	0.44 ± 0.16

Legends for Videos S1–S4

Video S1 – Live cells infected with PA-mNG PR8 virus for 10 hpi with MOI 0f 10 additionally treated with DMSO for 2h. Cells were imaged using OptiMEM or Leibovitz medium with a 63x oil immersion Nikon objective (NA = 1.4) on spinning disk confocal (SoRa) microscopes equipped with temperature (37ºC) and CO_2 (5%) regulated chamber and stage. Cells were imaged at 1 frame/ sec for 2 min using 488 nm laser for GFP. The video is part of Figure 2G.

Video S2 – Live cells infected with PA-mNG PR8 virus for 10 hpi with MOI 0f 10 and incubated with 5μM nucleozin for 2 hours. Cells were imaged using OptiMEM or Leibovitz medium with a 63x oil immersion Nikon objective (NA = 1.4) on spinning disk confocal (SoRa) microscopes equipped with temperature (37ºC) and CO_2 (5%) regulated chamber and stage. Cells were imaged at 1 frame/ sec for 2 min using 488 nm laser for GFP. The video is part of Figure 2G.

Video S3 – Volumetric animation of a fixed A549 cell infected with PA-mNG PR8 virus for 10 h showing viral inclusions on account of mNG fluorescence. Cells were imaged using OptiMEM or Leibovitz medium with a 63x oil immersion Nikon objective (NA = 1.4) on spinning disk confocal (SoRa) microscopes equipped with temperature (37ºC) and CO_2 (5%) regulated chamber and stage. Cells were imaged using a Z-stack and processed in Imaris.

Video S4 – Volumetric animation of a fixed A549 cell infected with PA-mNG PR8 virus for 10 h and treated with nucleozin for additional 2 hours showing viral inclusions on account of mNG fluorescence. Cells were imaged using OptiMEM or Leibovitz medium with a 63x oil immersion Nikon objective (NA = 1.4) on spinning disk confocal (SoRa) microscopes equipped with temperature (37ºC) and CO_2 (5%) regulated chamber and stage. Cells were imaged using a Z-stack and processed in Imaris.
